# Modest NT-proBNP Elevation in Septuagenarians Without Heart Failure Is Not Associated with Cardiac Alterations or Cardiovascular Outcomes

**DOI:** 10.3390/jcm14072407

**Published:** 2025-04-01

**Authors:** Cristina Oliveira da Silva, Camilla Hage, Jonas Johnson, Magnus Bäck, Anikó I. Nagy, Emma Svennberg, Larissa Bastos, Johan Engdahl, Faris Al-Khalili, Lars Lund, Aristomenis Manouras

**Affiliations:** 1 Department of Medicine, Karolinska Institutet, 17177 Stockholm, Sweden; 2 Heart and Vascular Center, Unit of Heart Failure, Arrhythmia and GUCH, Karolinska University Hospital, 17177 Stockholm, Sweden; 3 Heart and Vascular Center, Semmelweis University, 1085 Budapest, Hungary; 4 Department of Medicine Huddinge, Karolinska Institutet, 17177 Stockholm, Sweden; 5 Department Clinical Sciences, Karolinska Institutet, Danderyd Hospital, 17177 Stockholm, Sweden

**Keywords:** heart failure, atrial fibrillation, age, N-terminal pro-brain natriuretic peptide, echocardiography

## Abstract

**Background/Objectives:** To assess the association between moderate N-terminal natriuretic peptide (NT-proBNP) and cardiac alterations and prognosis in septuagenarians without heart failure (HF). **Methods:** From the STROKESTOP II screening study, 230 individuals aged 75/76 years with NT-proBNP < 900 ng/L were randomly selected. Subjects with persistent atrial fibrillation (AF), more than mild valvular disease, or HF were excluded. Echocardiography was performed. NT-proBNP ≥ 125 ng/L and paroxysmal AF (pAF) on thumb ECG were used as grouping variables. Participants were followed up during a median of 5 years for cardiovascular mortality, HF, AF, and cerebrovascular events. Cox regression analysis was employed for prognostic assessment. **Results:** Three groups were identified: SR ≥ 125 (*n* = 94, no pAF and NT-proBNP ≥ 125 ng/L), pAF (*n* = 77, pAF and NT-proBNP ≥ 125 ng/L), and controls (*n* = 30, no pAF and NT-proBNP < 125 ng/L). NT-proBNP was not associated with structural (left atrial volume and left ventricular (LV) mass) or functional (E/e’, LV strain) alterations in any group (*p* > 0.05). Cardiovascular risk factors (HR: 4.6; CI = 1.7–12.3; *p* = 0.002), but not NT-proBNP (HR: 1.9; CI = 0.7–5.1; *p* = 0.2), entailed a prognostic value for the composite endpoint of HF, AF, and cardiovascular death. **Conclusions:** In septuagenarians without HF, modest NT-proBNP elevation was not associated with echocardiographic changes or prognosis

## 1. Introduction

Measurement of N-terminal pro-B-type natriuretic peptide (NT-proBNP) is currently an inherent component of the clinical investigation workup for heart failure (HF). In recent European guidelines for HF, an NT-proBNP value of less than 125 ng/L has been endorsed as an indicator for ruling out non-acute HF [1]. Furthermore, there is clear interest in the role of NT-proBNP in risk assessments for cardiovascular morbidity in individuals without established HF [2]. NT-proBNP has also been investigated as a screening tool for HF in the general population [3,4]. Moreover, it has been shown that NT-proBNP levels correlate with echocardiographic functional and structural indices in patients with diastolic dysfunction [5], as well as in healthy individuals [6].

In the ambulatory setting, NT-proBNP is frequently employed as a straightforward method to identify underlying cardiac dysfunction, particularly among elderly individuals who lead sedentary lifestyles and seek medical attention for nonspecific symptoms. However, it has been observed that NT-proBNP levels tend to increase with age, primarily reflecting the burden of cardiovascular comorbidities in the elderly population [7]. Additionally, evidence suggests that age-specific changes in cardiac structure and function may contribute to the elevation of NT-proBNP levels [8]. Consequently, the interpretation of modest NT-proBNP elevations, particularly in elderly sedentary patients, presents a significant challenge.

The objective of our prospective study was to investigate whether modest elevations of NT-proBNP in asymptomatic septuagenarians without HF indicate subtle structural or functional cardiac alterations or have prognostic significance.

## 2. Methods

### 2.1. Study Population

The present investigation comprised a sub-study of a randomized population-based trial of AF screening (STROKESTOP II) described in a previous publication [9]. Briefly, the entire population of inhabitants, aged 75/76 years (*n* = 28,712), in Stockholm, Sweden, was randomized 1:1 to the intervention (AF screening) or control group (9). From the interventional arm of the study, participants without known AF (*n* = 6315) underwent an index-ECG, consisting of a 30 s ECG using a handheld one-lead device (Zenicor 2 device, Zenicor Medical Systems, Stockholm, Sweden) to detect asymptomatic/unknown AF [10,11], as well as an NT-proBNP analysis. NT-proBNP < 125 ng/L was found in 2549 (40.4%) participants, whereas values ≥ 125 ng/L were found in 3766 (59.6%) participants. NT-proBNP ≥ 900 ng/L was documented in 102 (1.6%) participants without known HF.

In the current study, 100 subjects with NT-proBNP 125–900 ng/L without evidence of pAF, 100 subjects with NT-proBNP 125–900 ng/L with pAF, and 30 subjects with NT-proBNP < 125 ng/L were digitally randomized using MS Excel from the screening arm of the STROKESTOP II study, after the exclusion of subjects with previously known heart failure. Subjects with more than mild valvular disease, permanent AF, HF, or prior cardiac surgery were excluded. The regional ethics committee approved this study. All participants received oral and written information and signed informed consent documents.

### 2.2. Assessment of Clinical Background and Medical Therapy

Data on HF symptoms and medical history, including diagnosis of angina, myocardial infarction, diabetes mellitus, and arterial hypertension, were collected using a self-questionnaire. Permanent AF was excluded by thumb ECG in subjects without an established diagnosis of AF. Classification of previous cardiovascular disease at recruitment was based on linked data from the Swedish National Patient Register using the International Classification of Disease, 10th revision (ICD-10), and self-reported questionnaires. Heart failure was defined on the basis of the triad of symptoms, structural echocardiographic changes, and NT-proBNP elevation. Brachial blood pressure was measured after 10 min of rest. The cardiovascular risk factor profile was assessed using the following variables: age, body mass index (BMI), and a diagnosis of hypertension, diabetes, or AF, according to the risk factors and findings consistent with heart failure with preserved ejection fraction in a symptomatic patient presented by the HFA–PEFF diagnostic algorithm [12].The participants were followed for a median (IQR) of 5 (4.8–5.5) years. Outcomes were obtained from medical records and the Swedish National Patient Register, and comprised the following: diagnosis of HF or AF, myocardial infarction, stroke/transient ischemic attack (TIA), and cardiovascular death.

### 2.3. Standard Echocardiography

All subjects underwent transthoracic echocardiography using a Vivid E9 ultrasound system (GE Healthcare). Offline analysis was performed by an experienced sonographer (>10 years of experience) using EchoPAC version 202 software (GE Healthcare), blinded to NT-proBNP levels.

Left ventricular (LV) dimensions were measured, and LV mass was calculated from the parasternal long-axis view using the following formula:

LV mass = 0.8 × [1.04 × (LVEDD + PWTd + SWTd)³ − (LVEDD)³] + 0.6,

where LVEDD = LV end-diastolic diameter, PWTd = end-diastolic posterior wall thickness, and SWTd = end-diastolic septal wall thickness. LV mass was indexed to body surface area to derive the LV mass index.

In the apical 2- and 4-chamber views, LV end-diastolic and end-systolic volumes were measured using the biplane method of disks, and EF was calculated. Tricuspid annular plane systolic excursion (TAPSE) was assessed in the four-chamber view using M-mode.

### 2.4. Speckle Tracking Echocardiography

Two-dimensional speckle tracking was performed to assess LV global longitudinal strain (GLS) using apical 4-chamber, 2-chamber, and long-axis views. All images were acquired at a frame rate of 60–80 frames per second. Aortic valve closure timing was determined by observing aortic valve motion in the apical long-axis view, and guided by ECG. For patients in sinus rhythm (SR), three consecutive cardiac cycles were analyzed.

Peak atrial longitudinal strain (PALS) was measured by manually tracing the endocardial border in apical 4- and 2-chamber views, using the QRS complex as the reference point. LA endocardial borders were delineated at the end-diastolic frame, defined by the QRS complex, and the region of interest was adjusted to include the entire LA wall thickness, while excluding the appendage and pulmonary veins. PALS was calculated as the peak value during the reservoir phase, represented as the average of all LA segments in the 4- and 2-chamber views. The frame rate was set between 60 and 80 frames per second.

Functional and morphological abnormalities were classified according to the consensus document by Pieske et al. [12].

### 2.5. Assessment of Diastolic Function

LV diastolic function was assessed using trans-mitral E and A flow velocities, the E/A ratio, E-wave deceleration time, and early diastolic myocardial velocities (e′) from the septal and lateral LV basal walls. From the apical 4-chamber view, pulsed-wave Doppler was applied at the tips of the mitral leaflets to measure peak early (E) and late (A) diastolic filling velocities, the E/A ratio, and E-wave deceleration time.

Tissue Doppler imaging of the mitral annulus was performed at the septal and lateral positions to obtain peak early (e′) velocities, from which the E/e′ ratio was calculated using the mean e′ value. The highest tricuspid regurgitation (TR) velocity was recorded and used to calculate the peak tricuspid regurgitation gradient via the modified Bernoulli equation. This was combined with the estimated right atrial pressure, derived from inferior vena cava collapsibility in the subcostal view during inspiration, to estimate pulmonary arterial systolic pressure.

LA volume was measured using the disks technique, and was indexed to body surface area (LAVi). LA enlargement was defined as an LAVi > 34 mL/m^2^. LV hypertrophy was defined as an LV mass index (LVMi) > 95 g/m^2^ in women and >115 g/m^2^ in men. All measurements were performed in accordance with current guidelines [13,14].

Diastolic function was classified according to the current recommendations using the E/é, e′ velocity, TR maximal velocity, and LAVi [14].

### 2.6. Natriuretic Peptides

NT-proBNP levels were measured at the Karolinska University Hospital core laboratory using a elecsys electrochemiluminescence ‘sandwich’ immunoassay proBNPII (Roche Diagnostics, Bromma, Sweden), with a lower detection limit of 5 ng/ L and interassay coefficients of variation of ≤20% [15]. The NT-proBNP threshold value of 125 ng/L was used for subgrouping, in accordance with current recommendations [1,9]. Since the HFA-PEFF diagnostic HFpEF workup proposes a cut-off value of NT-proBNP > 220 ng/L in patients without AF [12], this threshold value was also used as a grouping value and tested separately.

## 3. Statistical Analysis

Normality was assessed using skewness and histograms. Continuous variables were presented as the mean ± standard deviation (SD), or as the median and interquartile range (IQR), based on their distribution (Gaussian or non-Gaussian); percentages were used for discrete variables. In order to assess inter-observer variability, two sonographers (with more than five years of experience) performed the exact same procedure of measuring PALS in 20 subjects in a blinded manner. One of the sonographers repeated the measurements in a randomly selected order on a different day, in order to determine the intra-observer variability. The agreement between observers was evaluated using the Intraclass Correlation Coefficient (ICC) to assess consistency, and a Bland–Altman analysis to determine the limits of agreement.

Comparisons between two groups were assessed using the *t*-test or the Mann–Whitney test, as appropriate. Comparisons between more than two groups were assessed using one-way analysis of variance (ANOVA) for normally distributed continuous variables, and the Kruskal–Wallis test when the normality assumptions were not satisfied. The Chi-square test was used to compare categorical variables, and Fisher’s exact test was employed when the chi-square assumptions were not met. Correlations between continuous normally distributed variables were assessed using Pearson’s correlation coefficient, whereas Spearman’s rank correlation was used for non-normally distributed variables.

Univariate and multivariate Cox proportional models were used to assess the association of echocardiographic variables and NT-proBNP levels with the composite outcome of HF, AF, myocardial infarction, stroke/TIA, and cardiovascular death. A risk factor profile was also included in the analysis to account for potential confounders. In the multivariate model, E/é > 14, LAVi > 34 mL/m^2^, GLS ≤ 18%, PALS < 18%, >2 risk factors, LVMi, and NT-proBNP > 220 ng/mL were included.

The results for predictors are presented as hazard ratios (HRs) and 95% confidence intervals (CIs). The collected data were analyzed using SPSS version 21.0 for Windows (SPSS Inc., Chicago, IL, USA). Statistical significance was set at *p* < 0.05.

## 4. Results

### 4.1. Subject Characteristics

All the subjects were asymptomatic. Table 1 outlines the baseline characteristics and medication details of the participants. Based on an NT-proBNP cut-off value of 125 ng/L and evidence of pAF at screening, three groups were identified. After applying the exclusion criteria, the first group (elevated NT-proBNP (125–900 ng/L and no pAF) comprised 94 participants, and was denoted as SR ≥ 125. The second group, pAF (NT-proBNP 125–900 ng/L and documented pAF at screening), consisted of 77 subjects. The control group (NT-proBNP < 125 ng/L and no pAF) included 30 individuals. None of the participants with evidence of pAF at screening had an NT-proBNP level of <125 ng/L. A flowchart of the inclusion process is presented in Supplementary Figure S1.

There were more females in the group than in the pAF and control groups (*p* = 0.005). There were no significant differences in NT-proBNP levels between sexes across all groups (*p* > 0.05). The prevalence of comorbidities was similar among the three groups.

The echocardiographic characteristics are summarized in Table 2. Compared with both the SR ≥ 125 and control groups, the pAF group had a significantly higher LAVi, a lower stroke volume, and reduced GLS (*p* < 0.001 for all, except GLS: *p* = 0.015 vs. SR ≥ 125 and *p* = 0.011 vs. controls). TR velocity was also higher in the pAF group compared with controls (*p* = 0.003). No significant differences were observed among the three groups in E/e′ mean ratio, LVMi, or LVEF. PALS did not differ significantly between the SR ≥ 125 and control groups.

To further investigate NT-proBNP as a classifier, participants without pAF were stratified, using the HFA-PEFF diagnostic algorithm cut-off of NT-proBNP ≥ 220 ng/L, into SR > 220 (NT-proBNP > 220 ng/L, no pAF; *n* = 45) and control220 (NT-proBNP < 220 ng/L, no pAF; *n* = 79). Comparisons between these groups yielded no substantial differences from the primary analysis (Supplementary Tables S1 and S2).

**Figure 1:**
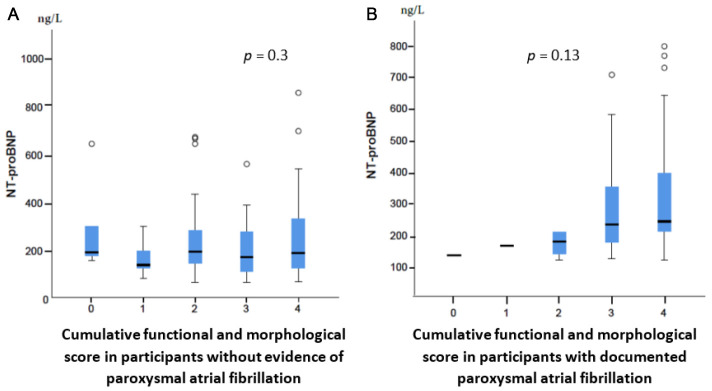
NT-proBNP levels in patients without and with morphological and functional abnormalities. Cumulative score of cardiac abnormalities was calculated as recommended by Pieske et al. (**A**) Cohorts without paroxysmal atrial fibrillation (pAF), (**B**) patients with pAF [12].

Morphological and functional cardiac abnormalities were assessed using a scoring system suggested by the HFA-PEFF algorithm. In subjects without evidence of pAF, no differences in morphological and functional changes we observed between those with normal and elevated NT-proBNP levels. Furthermore, NT-proBNP levels were similar between subjects with and without morphological and functional abnormalities (*p* > 0.05). In contrast, subjects with pAF displayed significantly higher scores of morphological and functional alterations, although NT-proBNP levels were not significantly higher in those with more alterations (Figure 1 and Supplementary Table S3).

### 4.2. Diastolic Function Assessment

**Figure 2:**
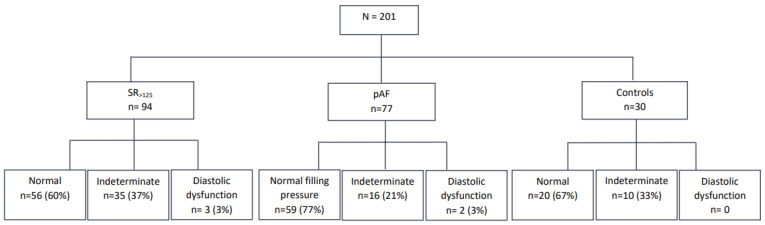
Diastolic function and filling pressure assessment.

Figure 2 illustrates the classification of diastolic function and filling pressures (15). In the SR ≥ 125 group, 56 subjects (60%) exhibited normal diastolic function, while 3 (3%) had diastolic dysfunction. Diastolic function was indeterminate in 34 subjects (37%), due to missing parameters (*n* = 15) or an equal number of positive and negative echocardiographic criteria (*n* = 19).

In the control group, diastolic function was indeterminate in 10 subjects (33%), while the remaining 20 (67%) had normal diastolic function. In the pAF group, 2 subjects (3%) had estimated high filling pressures, while 16 (21%) could not be assessed for filling pressures.

There were no significant differences in NT-proBNP levels across the three categories of diastolic function (normal diastolic function, 204 ± 15 ng/L; diastolic dysfunction, 326 ± 98 ng/L; indeterminate, 238 ± 25 ng/L; *p* = 0.24).

Diastolic function and filling pressures were determined according to the 2016 ASE/ECAVI recommendations for the evaluation of diastolic function by echocardiography.

### 4.3. Risk Factor Burden

In the entire cohort (N = 201), individuals with hypertension exhibited significantly higher NT-proBNP levels than those with normotension (hypertension: 226 [170–346] ng/L, *n* = 94, vs. normotension 184 [130–279] ng/L, *n* = 107; *p* = 0.006). A similar pattern was observed in the subgroup without pAF (hypertension, 180 ng/L [141–325 ng/L, *n* = 55] vs. normotension, 154 ng/L [106–265] ng/L, *n* = 69; *p* = 0.03). No significant difference was found in NT-proBNP levels between participants with BMI ≥ 30 kg/m^2^ (231 [147–349] ng/L, *n* = 32) and those with BMI < 30 kg/m^2^ (200 [141–299] ng/L, *n* = 169; *p* = 0.31). Similar results were observed when the analysis was confined to participants without pAF (BMI ≥ 30 kg/m^2^, 172 [135–287] ng/L, *n* = 104, vs. BMI < 30 kg/m^2^ 185 [123–277] ng/L, *n* = 20; *p* = 0.70)].

In the entire cohort (N = 201), the risk factor burden was assessed based on five factors: BMI ≥ 30 kg/m^2^, AF, diabetes mellitus, age > 70 years, and hypertension [12]. By study design, all participants had age as a risk factor. In total, 60 subjects had only age as a risk factor; two risk factors were documented in 79 participants, three in 49, four in 9, and five in 4 subjects. As shown in Table 3, subjects with more than one risk factor had significantly higher NT-proBNP levels (*p* < 0.001). Additionally, there were notable disparities in LVMi, LV GLS, LV end-diastolic diameter, and LAVi between subjects with one risk factor and those with multiple risk factors. When only participants without pAF were considered, significant differences were observed in NT-proBNP levels, LVMi, LV GLS, and LV end-diastolic diameter between those with one risk factor and those with multiple risk factors.

### 4.4. Outcome

During follow-up in the SR ≥ 125 group, four subjects died (two due to cardiac disease); eight developed AF, of whom two were also diagnosed with HF; and five subjects experienced myocardial infarction or stroke/TIA. In the pAF group, six subjects developed HF, two of whom also had myocardial infarction or stoke/TIA, and one died due to HF. Additionally, seven subjects experienced myocardial infarction or stoke/TIA. Finally, in the control group, one subject developed AF, one developed HF, and one was diagnosed with both AF and HF.

In subjects without evidence of pAF, the predictive value of E/e′, LAVi, LV GLS, PALS, LVMi, NT-proBNP, and risk factor burden (>1 and >2 additional risk factors) was assessed (Table 4). The presence of at least two additional cardiovascular risk factors beyond age was the only parameter significantly associated with prognosis in both univariate (HR 4.0; 95% CI 1.6–9.9, *p* = 0.003) and multivariate analysis (HR 4.1; 95% CI 1.6–10.5, *p* = 0.003).

### 4.5. Inter- and Intra-Observer Variability

Very good agreement between the two experienced sonographers was present in measuring LA strain, with an ICC of 0.88 (95% CI: 0.735 to 0.950). Bland–Altman analysis also showed low inter-observer variability (Supplementary Figure S2). Inter-observer analysis demonstrated excellent agreement, as shown by an ICC of 0.97 (95% CI: 0.929–0.988) and Bland–Altman plots (Supplementary Figure S3).

## 5. Discussion

In this screening cohort of asymptomatic septuagenarians without overt HF, we demonstrated that a modest NT-proBNP increase was not linked to echocardiographic structural or functional changes. Although NT-proBNP levels exceeding 220 ng/L were associated with a higher burden of cardiovascular risk factors, they failed to demonstrate significant prognostic value for cardiovascular events. In contrast, the occurrence of more than two risk factors exhibited strong prognostic value.

### 5.1. Association Between NT-proBNP Level and Echocardiographic Indices

The relationship between NT-proBNP levels and echocardiographic abnormalities in mild diastolic dysfunction, particularly in individuals without apparent HF symptoms, is still not fully understood. There is conflicting evidence as to whether NT-proBNP levels correspond to observable echocardiographic abnormalities in this context. For instance, Nah et al. did not find a significant association between diastolic dysfunction, as defined by E/e′ and e′ and circulating NT-proBNP levels in individuals without HF symptoms [6]. However, it is worth noting that Nah et al. did observe an association between left LVMi and NT-proBNP levels in their cohort [6]. Similarly, Abhayaratna et al. reported that NT-proBNP levels and structural cardiac alterations did not significantly differ between elderly individuals with mild diastolic dysfunction and age-matched controls [16]. In the current study, we did not observe significant differences in NT-proBNP levels across various degrees of structural/functional alternations when considering the entire cohort or when separately studying the subgroups without pAF. Nevertheless, it is important to acknowledge that none of the aforementioned studies, including ours, have assessed cardiac function during exertion. Therefore, the differential response of conventional echocardiographic indices to increased metabolic demands in this cohort remains unclear, and warrants further investigation.

In subjects with pAF, a modest correlation was observed between NT-proBNP levels and PALS (r = −0.27, *p* = 0.023). However, no significant correlation was observed between NT-proBNP levels and PALS in the other two groups (*p* > 0.05). Previous studies have reported an inverse association between PALS and NT-proBNP levels in various clinical conditions [17,18,19,20]. However, few studies have specifically investigated the relationship between PALS and NT-proBNP levels in asymptomatic individuals. For instance, Liu et al. found a poor correlation between PALS and NT-proBNP levels in a community-based population [21], whereas another study in elderly patients without HF showed a stronger association between PALS and NT-proBNP [22]. Notably, PALS was found to have significant prognostic value for the composite outcome of heart failure and death during a 5.5-year follow-up in the latter study. It is important to consider that our cohort excluded patients with chronic AF and significant valvular disease. This difference in patient characteristics between studies may contribute to the divergent findings, as the presence of AF can significantly influence the range and variability of both NT-proBNP and PALS values, thus impacting the observed degree of correlation, as well as the incidence of HF.

### 5.2. Association Between Risk Factors and NT-proBNP Levels

NT-proBNP levels increase with age. In a recent cross-sectional study including roughly 20,000 individuals without previous cardiovascular disease, 60% of female participants and 40% of male participants demonstrated an NT-proBNP value larger than 125 ng/L, with systematically higher values documented with increasing age [7]. Similar findings were reported in a long-term longitudinal study by Luchner et al., which showed age-associated increases in NT-proBNP levels, particularly in individuals aged >70 years [23]. Although poorly understood, this association might be attributed to aging-associated processes such as fibrosis [24]. Moreover, increased wall stress has been shown to yield higher NT-proBNP levels in patients with hypertension, which is the most common cause of increased LV wall stress [25]. Rivera et al. showed that hypertensive subjects had higher NT-proBNP levels than normotensive subjects. However, this difference disappeared when subjects with systolic and/or diastolic dysfunction were excluded from the analysis [26]. We also observed that subjects with arterial hypertension exhibited significantly higher NT-proBNP levels than those with normotension did. Importantly, only few individuals in our study exhibited diastolic dysfunction. Larger studies are needed to understand the possible association between NT-proBNP and diastolic function in elderly asymptomatic hypertensive subjects.

Apart from the inherent age-related processes, increased NT-proBNP can, at least in part, be ascribed to the underlying cardiovascular risk factor burden [7,27]. Consistent with these findings, we observed that the presence of multiple risk factors was associated with higher NT-proBNP levels. Furthermore, we demonstrated that the burden of cardiovascular risk factors was also reflected in a higher incidence of structural cardiac changes, even after excluding subjects with pAF.

### 5.3. Prognostic Value of NT-proBNP

In this cohort, NTproBNP elevation was not associated with the composite outcome of cardiovascular events and cardiovascular death, while the risk factor burden had significant prognostic value. This might suggest that the lack of NTproBNP prognostic ability might be attributed to the limited cohort or the narrow NT-proBNP range investigated in the current study, as it was specifically designed to clarify the clinical significance of moderately elevated NT-proBNP, particularly in subjects without HF or relevant valvular heart disease. Our findings are in contrast with previous findings. Rudolph et al., in a large cohort of elderly patients in the primary care setting, reported that higher NT-proBNP levels were significantly associated with cardiovascular and all-cause mortality, independently of traditional risk factors [28]. Another study also showed that NT-proBNP predicted mortality and HF in a general population free of overt HF in a nine-year follow-up study [29]. However, in the latter study, patients with reduced EF and valvular disease were not excluded. Furthermore, in the study by Rudolph et al., echocardiography was not performed, and significant structural or functional cardiac disease was not ruled out. The occurrence of more than two risk factors was an independent predictor of cardiovascular mortality and cardiovascular events combined, supporting previous studies that have shown an association between cardiovascular risk factors and the development of cardiovascular events and death [30,31,32,33,34].

## 6. Limitations

This study has several limitations. The sample size was limited, and filling pressures were not confirmed using invasive measurements. Additionally, all examinations were performed at rest, raising uncertainty regarding whether the assessed diastolic indices would demonstrate any pathological changes in response to increased metabolic demand. Reduced renal function is a known cause of elevated natriuretic peptide levels [35]. Plasma creatinine levels were not recorded in our study, preventing adjustment for renal function in relation to NT-proBNP levels. However, only a small number of participants (*n* = 7) had chronic kidney disease, with no significant differences in its prevalence between groups. Furthermore, as the diagnosis of pAF was made based on a 30 s thumb ECG, the presence of undiagnosed cases cannot be excluded.

## 7. Conclusions

In elderly asymptomatic subjects without a diagnosis of HF, moderate elevations in NT-proBNP levels were not associated with overt morphological or functional cardiac changes at rest, particularly in the absence of AF. Furthermore, NT-proBNP did not carry any significant prognostic value in this screening cohort. Instead, the presence of more than two cardiovascular risk factors provided important prognostic insight into cardiovascular morbidity and mortality. These findings suggest that in elderly patients, moderately elevated NT-proBNP levels do not necessarily indicate cardiac disease. Rather, comprehensive risk factor profiling provides more reliable diagnostic and prognostic information.

## Figures and Tables

**Table 1:** Baseline characteristics and medical history of cohort.

	SR ≥ 125 (*n* = 94)	pAF (*n* = 77)	Controls (*n* = 30)	*p*-Value
Male n (%)	31 (33)	43 (56)	17 (57)	0.005
HR (beats/min)	67± 11	69 ± 12	68 ± 9	0.56
SBP (mmHg)	140 ± 16 *	135 ± 19	137 ± 17	0.14
DBP (mmHg)	77 ± 11	80 ± 12	79 ± 11	0.18
BMI (kg/m^2^)	24 (22–28)	26 (23–28)	25 (23–28)	0.49
NT-proBNP (ng/L)	217 (156–317) **	235 (183–376) **	93 (79–103)	<0.001
Comorbidities				
Hypertension, n (%)	46 (49)	39 (51)	9 (30)	0.13
Hyperlipidemia, n (%)	12 (13)	11 (14)	4 (13)	0.96
Diabetes mellitus, n (%)	10 (11)	7 (9.)	3 (10)	0.95
Previous stroke/TIA, n (%)	7 (7)	2 (3)	2 (6.7)	0.33
Coronary artery disease, n (%)	7 (7)	3 (4)	0	0.17
Peripheral artery disease, n (%)	3 (3)	1 (1)	2 (7)	0.37
Chronic kidney disease, n (%)	2 (2)	5 (7)	-	0.11
COPD	1 (1)	4 (5)	2 (7)	0.17
Medication				
ACEi/Ang II, n (%)	33 (35)	37 (48)	6 (20)	0.32
Statins, n (%)	25 (27)	15 (20)	9 (30)	0.41
CCB, n (%)	23 (25) *	17 (22)	2 (7)	0.11
Diuretics, n (%)	12 (13)	12 (16)	4 (13)	0.87
Β-blockers, n (%)	21 (22)	23 (30)	4 (13)	0.18

Categorical variables are expressed as frequencies (percentages), and continuous variables as mean ± SD, n (%), or median (25th to 75th percentiles). * denotes significant differences compared to control group at a significance level of 0.05. ** denotes significant differences compared to control group at a significance level of 0.001. HR, heart rate; SBP; systolic blood pressure; DBP, diastolic blood pressure; BMI, body mass index; TIA, transient ischemic attack; COPD, chronic obstructive pulmonary disease; ACEi, angiotensin converting enzyme inhibitor; Ang II, angiotensin II receptor inhibitor; CCB, calcium channel blocker.

**Table 2:** Echocardiographic characteristics of study subjects.

	SR ≥ 125 (*n* = 94)	pAF (*n* = 77)	Control (*n* = 30)	*p*-Value
LVEDD (mm)	44.5 ± 4.6	45.9 ± 5.0	44.9 ± 3.7	0.13
Septal thickness (mm)	9.5 ± 1.2	9.6 ± 1.5	9.7 ± 1.2	0.74
LVMi (g/m^2^)	73.6 ± 15.1	77.2 ± 17.9	71.9 ± 12.9	0.50
LV hypertrophy, n (%)	4 (4.3)	4 (5.2)	1 (3.3)	0.91
LV EF (%)	60.3 ± 5.5	59.2 ± 5.8	60 ± 5.0	0.51
TAPSE (mm)	23.7 ± 4.0 (93)	24.5 ± 3.9	23.6 ± 4.1	0.27
TR Vmax (m/s)	2.5 ± 0.3 (70)	2.6 ± 0.3 (63) *	2.4 ± 0.25 (16)	**0.04**
E/é mean	10.2 ± 2.9	9.9± 3.0 (62)	10.0 ± 2.9	0.28
E/A ratio	0.9 ± 0.2	0.9 ± 0.3 (67)	0.9 ± 0.4	0.32
Stroke volume (mL/m^2^)	46.0± 8.2	37.6 ± 8.6 ††**	43.5 ± 12.5	**<0.001**
LAVi (mL/m^2^)	29.0 ± 7.4 (93)	37.4 ± 9.3 **††	28.4 ± 6.2	**<0.001**
LA enlargement, n (%)	21 (22%)	47 (61%) **††	6 (20%)	**<0.001**
LV GLS (%)	20.0 ± 2.2 (90)	18.8 ± 2.6 (57) *†	20 ± 2.4	**0.01**
PALS (%)	30.7 ± 7.6	NA	31.4 ± 9.7	0.71

Values are presented as mean ± SD or frequencies and percentages. * denotes significant differences compared to control group at the significance level of 0.05. **, significant differences compared to control group at the significance level of 0.001. † denotes significant differences compared to SR ≥ 125 at the significance level of 0.05. †† denotes significant differences compared to SR ≥ 125 at the significance level of 0.001. (*n*), number of participants included in analysis. LVEDD, left ventricular end-diastolic diameter; LVMi, left ventricular mass index; LV EF, left ventricular ejection fraction; TAPSE, tricuspid annular plane systolic excursion; TRVmax, tricuspid regurgitation maximal velocity; é, early diastolic mitral annular tissue velocity; E/A ratio, early to late diastolic transmitral flow velocity; LAVi, left atrial volume index; GLS, global longitudinal strain; PALS, peak atrial longitudinal strain. LV hypertrophy was defined as LVMi > 95 or 115 g/m^2^ in females and males, respectively; LA enlargement was defined as LAVi > 34 mL/m^2^.

**Table 3:** Association of risk factor profile with NT-proBNP levels and echocardiographic variables.

	SR ≥ 125 and Control Group		*p*-Value	Entire Cohort		*p*-Value
	Risk Factor =1 (*n* = 60)	Risk Factors > 1 (*n* = 64)		Risk Factor =1 (*n* = 60)	Risk Factors > 1 (*n* = 141)	
NT-proBNP (ng/mL)	154 (104–245)	189 (141–325)	0.024	154 (104–245)	221 (164–349)	<0.001
LVMi (g/m^2^)	68.5 ± 12.8	79.1 ± 15.6	<0.001	68.5 ± 12.8	78.1 ± 16.9	<0.001
LV GLS (%)	20.7 ± 2.2 (58)	19.4 ± 2.2 (58)	0.06	20.7 ± 2.2 (58)	19.1 ± 2.4 (115)	<0.001
LAVi (mL/m^2^)	28 ± 6	30 ± 8	0.26	28 ± 6	34 ± 10	<0.001
LV EDD (mm)	43.2 ± 4.1	46.0 ± 1.1	<0.001	43.2 ± 4.1	45.9 ± 4.7	0.001

SR ≥ 125 denotes the group of participants with NTproBNP ≥ 125 ng/L and no evidence of paroxysmal atrial fibrillation at screening. Control group refers to the group of participants with NT-proBNP < 125 ng/L and no evidence of pAF at screening. Entire cohort denotes the entire cohort, i.e., participants with SR ≥ 125 and the control group, as well as the pAF group. Risk factor = 1 refers to participants with age as only risk factor. Risk factors > 1 refers to participants with additional risk factors to age. LVMi, left ventricular mass index; LV GLS, left ventricular global longitudinal strain; LAVi, left atrial volume index; LVEDD, left ventricular end-diastolic diameter.

**Table 4:** Univariate and multivariate analysis of association of NT-proBNP levels and composite outcome of heart failure, atrial fibrillation, myocardial infarction, stroke/TIA, and cardiovascular death in subjects without evidence of paroxysmal atrial fibrillation.

SR ≥ 125 and Control Group	Univariate Analysis			Multivariate Analysis		
	HR	95% CI	*p*-Value	HR	95% CI	*p*-Value
E/é mean > 14	1.0	0.2–4.3	0.98	0.8	0.2–3.7	0.78
LAVi > 34 (mL/m^2^)	0.9	0.3–2.8	0.90	1.0	0.3–3.3	0.95
LV GLS ≤ 18 (%)	1.3	0.5–3.5	0.85	1.7	0.6–5.0	0.34
Risk factor > 1	2.0	0.8–5.3	0.16	-	-	**-**
Risk factor > 2	4.0	1.6–9.9	**0.003**	4.6	1.7–12.3	**0.002**
NT pro BNP ≥ 125 (ng/L)	1.0	0.6–1.8	0.79	-	-	-
NT pro BNP > 220 (ng/L)	2.0	0.8–5.0	0.12	1.9	0.7–5.0	0.22
PALS < 18 (%)	4.0	0.9–17.6	0.06	2.6	0.5–13.4	0.26
LVMi (mg/m^2^)	1.0	0.99–1.05	0.19	1.8	0.7–4.8	0.27

E/é mean: early diastolic mitral annular tissue velocity to early diastolic transmitral flow velocity; LAVi: left atrial volume index; LV GLS: left ventricular global longitudinal strain; PALS: peak atrial longitudinal strain; LVMi, left ventricular mass index. LVMi was used as continuous variable adjusted for gender.

## Data Availability

The data presented in this study are available on request from the corresponding author due to privacy reasons.

## Supplementary Materials

The following supporting information can be downloaded at https://www.mdpi.com/article/10.3390/jcm14072407/s1: Figure S1: Flowchart of patient selection; Figure S2: Bland-Altman plots for inter-observer variability between the two sonographers (1 vs. 2). PALS: peak systolic atrial strain; SD: standard deviation; Figure S3. Bland-Altman plots showing intra-observer variability for measurement 1 and 2 for the same sonographer. PALS: peak systolic atrial strain; SD: standard deviation; Table S1: Baseline characteristics and medical history in participants without paroxysmal atrial fibrillation dichotomized in 2 groups based on NT-proBNP threshold of 220 ng/L; Table S2: Echocardiographic characteristics in participants without paroxysmal atrial fibrillation dichotomized in 2 groups based on NT-proBNP threshold of 220 ng/L; Table S3: Grading of structural and functional abnormalities by echocardiography.

## Abbreviations

AF	atrial fibrillation
CI	confidence interval
EF	ejection fraction
GLS	global longitudinal strain
HF	heart failure
HR	hazard ratio
IQR	interquartile range
LAVi	left atrial volume index
LV	left ventricle
NT-proBNP	N-terminal pro B-type natriuretic peptide
pAF	paroxysmal AF
PALS	peak left atrial reservoir strain
TAPSE	tricuspid annular plane systolic excursion
TRVmax	tricuspid regurgitation maximal velocity
SR	sinus rhythm
TIA	transient ischemic attack
