# Supplementary material for: Modest NT-proBNP Elevation in Septuagenarians Without Heart Failure Is Not Associated with Cardiac Alterations or Cardiovascular Outcomes
Source: J Clin Med. 2025 Apr 1;14(7):2407. doi: 10.3390/jcm14072407 (PMC11989729; doi:10.3390/jcm14072407)
Supplement: Supplementary file 1 [file jcm-14-02407-s001.zip › jcm-3487643-supplementary.pdf]

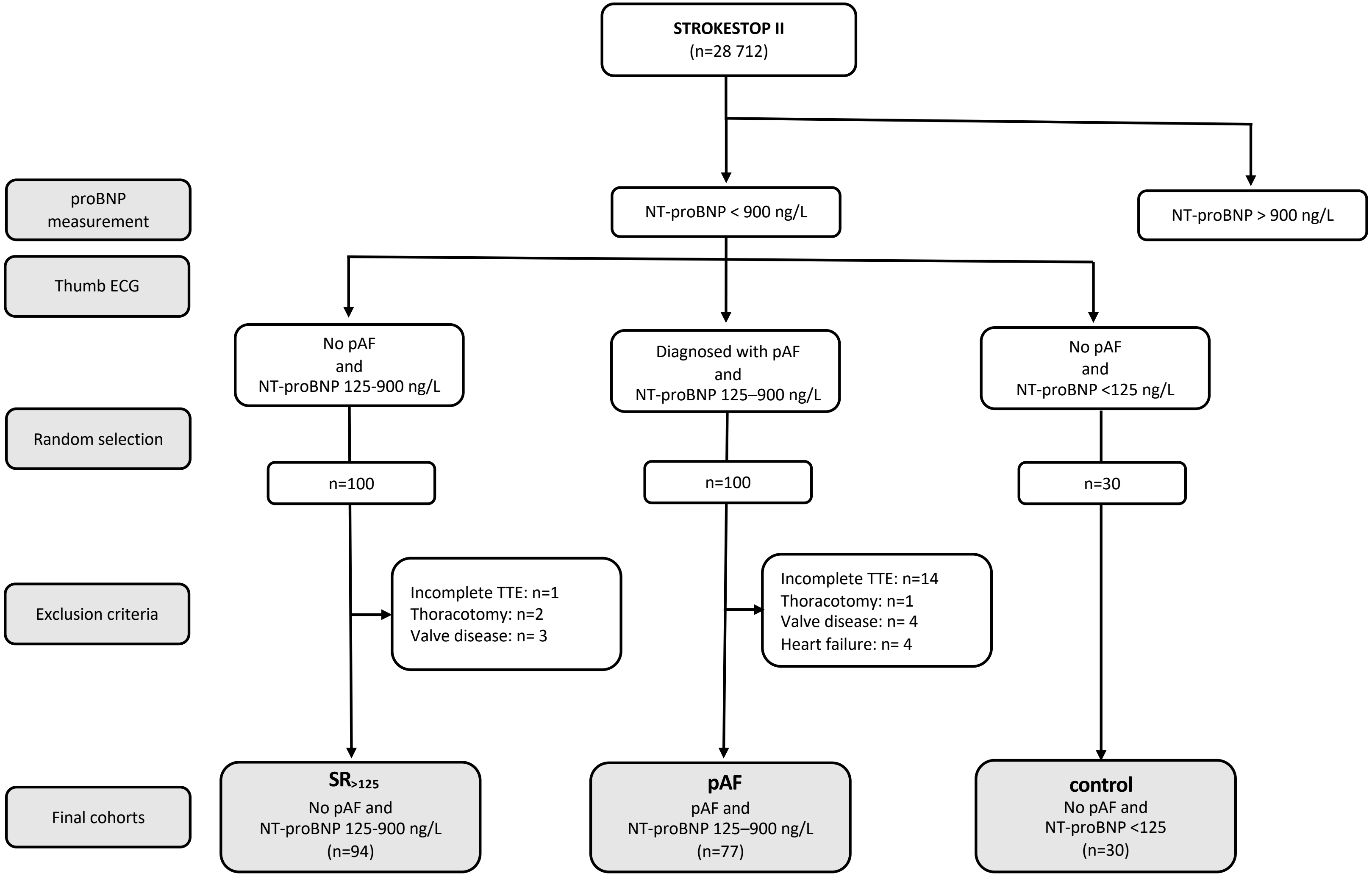

Supplementary Figure S1. Flowchart of patient selection. More than mild valve disease was considered an exclusion criterion. pAF, paroxysmal atrial fibrillation

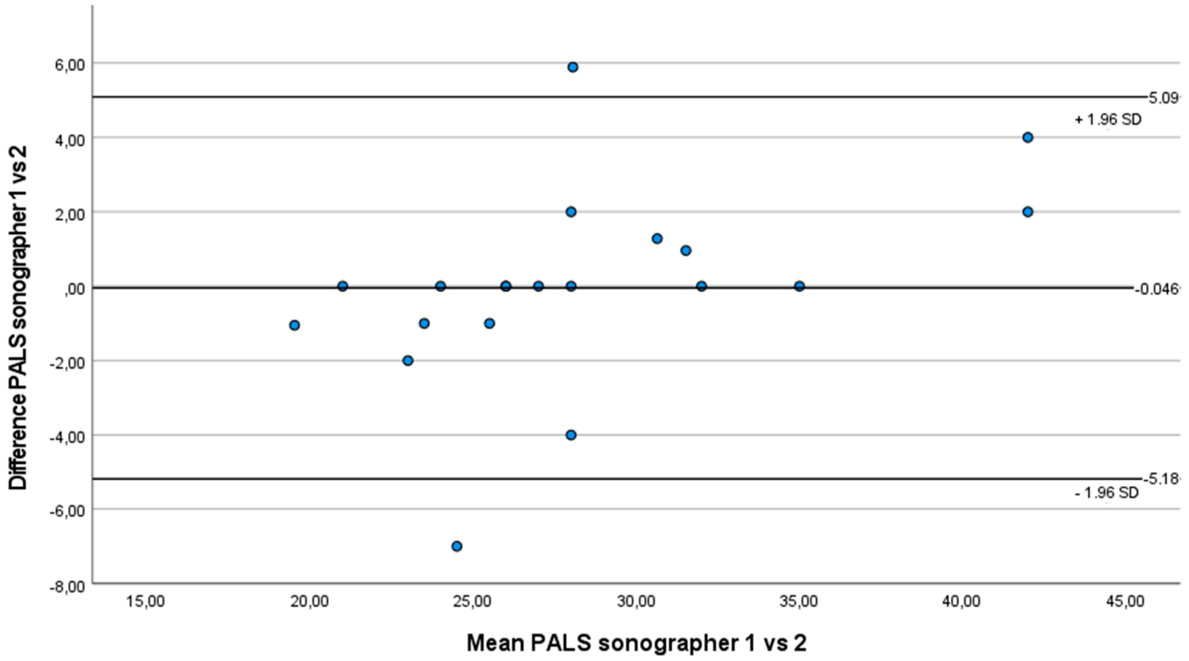

Supplementary Figure S2. Bland-Altman plots for inter-observer variability between the two sonographers (1 vs 2). PALS: peak systolic atrial strain; SD: standard deviation.

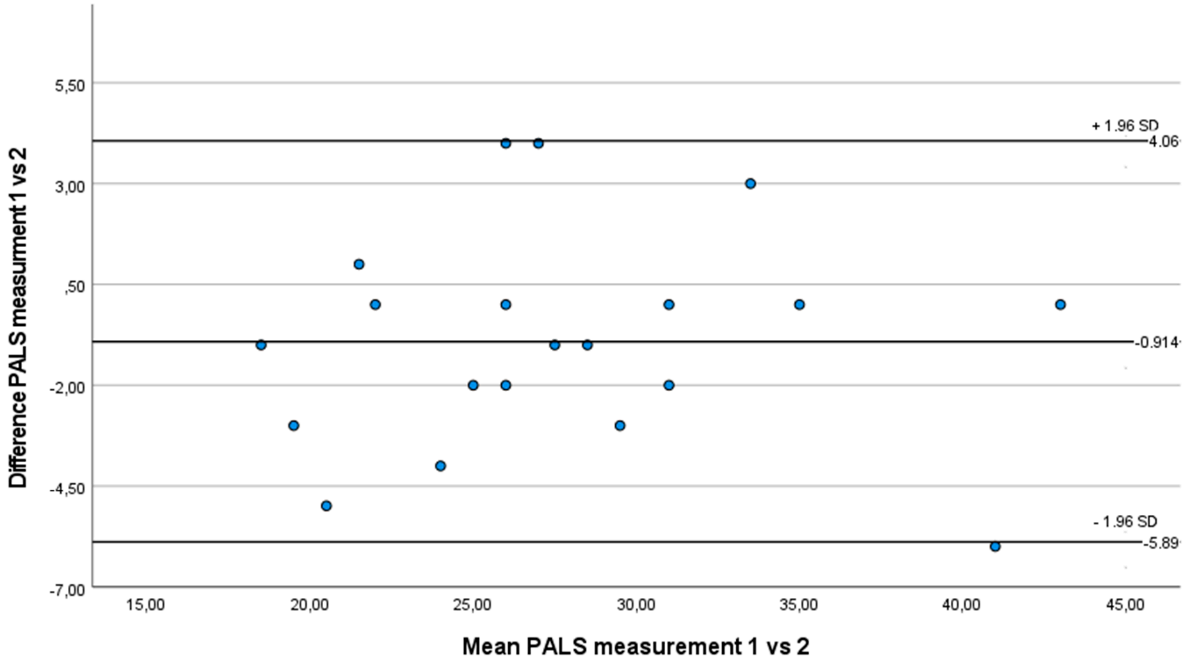

Supplementary Figure S3. Bland-Altman plots showing intra-observer variability for measurement 1 and 2 for the same sonographer. PALS: peak systolic atrial strain; SD: standard deviation.

Supplementary Table S1. Baseline characteristics and medical history in participants without paroxysmal atrial fibrillation dichotomized in 2 groups based on NT-proBNP threshold of 220 ng/L

	$SR_{>220}$ (45)	$Controls_{>220}$ (n=79)	p-value
Male n (%)	13 (29)	35 (44)	0.13
HR (beats/min)	67± 11	68 ± 10	0.75
SBP (mmHg)	140± 15	137 ± 17	0.15
DBP (mmHg)	78 ± 12	78 ± 10	0.71
BMI (kg/m^2)	25 ± 4	26 ± 4	0.16
NT-proBNP (ng/L)	317 (275 - 386)	141 (97 - 172)	<0.001
Comorbidities			
Hypertension, n (%)	22 (49)	33 (42)	0.46
Hyperlipidemia, n (%)	7 (16)	9 (11)	0.59
Diabetes mellitus, n (%)	5 (11)	8 (6)	0.54
Previous stroke/TIA, n (%)	1 (2)	8 (10)	0.15
Coronary artery disease, n (%)	4 (9)	3 (4)	0.25
Peripheral Artery disease, n (%)	1 (2)	4 (5)	0.65
Chronic kidney disease, n (%)	2 (4)	0	0.13
COPD	1	2 (3)	0.7
Medication			
ACEi/Ang II, n (%)	16 (36)	23 (29)	0.58
Statins n (%)	10 (22)	24 (29)	0.4
CCB, n (%)	11 (24)	14 (30)	0.49

Diuretics, n (%)	7 (16)	9 (11)	0.58
B-blockers, n (%)	14 (31)	11 (14)	0.035

Table Legend: Categorical variables are expressed as frequencies (percentages). Continuous variables as mean ± SD, n (%), or median (25th to 75th percentiles). HR, heart rate; SBP; systolic blood pressure; DBP, diastolic blood pressure; BMI, body mass index; TIA, transient ischemic attach; COPD, chronic obstructive pulmonary disease; ACEi, angiotensin converting enzyme inhibitor; Ang II, angiotensin II receptor inhibitor; CCA, calcium channel blocker.

Supplementary Table S2. Echocardiographic characteristics in participants without paroxysmal atrial fibrillation dichotomized in 2 groups based on NT-proBNP threshold of 220 ng/L

	$SR_{>220}$ (n = 45)	$Control_{>220}$ (n = 79)	p-value
LVEDD (mm)	44.3 ± 3.9	44.5 ± 4.7	0.98
Septal thickness (mm)	9.7 ± 1.0	9.5 ± 1.3	0.70
LVMi (g/m^2)	74.7 ± 14	74.0 ± 16.6	0.81
LVEF (%)	60 ± 6	60 ± 5	0.99
TAPSE (mm)	25.2 ± 3.4	23.6 ± 3.8 *(78)*	0.33
TR Vmax (m/s)	2.6 ± 0.3 *(34)*	2.4 ± 0.3 (59*)*	0.01
é mean (cm/s)	7.9 ± 1.6	7.6 ± 1.6	0.43
E/é mean	9.9 ± 2.8	10.5 ± 3.0	0.21
E/A	0.9 ± 0.3	0.9 ± 0.2	0.62
Stroke volume index (ml/m^2)	46 ± 8	46 ± 9	0.46

LAVi (ml/m^2)	30 ± 7	28 ± 7 (78)	0.13
LV GLS (%)	20.0 ± 2.1 *(44)*	31.8 ± 8.7 (72)	0.73
PALS (%)	24.4 ± 7.2 (43)	31.4 ± 9.7 (75)	0.23

Table Legend: Values are presented as the mean ± SD or frequencies and percentages. LVEDD, left ventricular end-diastolic diameter; LVMi, LV mass index; LV EF, left ventricular ejection fraction; TAPSE, tricuspid annular plane systolic excursion; TRVmax, tricuspid regurgitation maximal velocity; é, early diastolic mitral annular tissue velocity; E/A ratio, early to late diastolic transmitral flow velocity; LAVi, Left atrial volume index; LV GLS, left ventricular global longitudinal strain; PALS, peak atrial longitudinal strain. LV hypertrophy was defined as LVMi > 95 or 115 g/m^2 in females and males, respectively; LA enlargement was defined as LAVi >34 ml/m^2.

Supplementary Table S3. Grading of structural and functional abnormalities by echocardiography

Total score	$SR_{>125}$	pAF	Control	p ($SR_{>125}$ vs. control)	p (pAF vs without pAF)
0 point	5 (5%)	1 (1%)	-	0.33	0.41
1 point	8 (9%)	1 (1%)	3 (10%)	0.73	0.032
2 points	33 (35%)	6 (8%)	9 (30%)	0.17	0.0001
3 points	30 (32%)	24 (31%)	12 (40%)	0.50	0.76
4 points	18 (19%)	45 (58%)	6 (20%)	1.0	0.0001

Table Legend

Classification of the structural and functional alterations based on the HFA-PEFF diagnostic algorithm (Pieske et al. reference nr 12). $SR_{>125}$ denotes the groups of participants without evidence of paroxysmal atrial fibrillation (pAF) with NT-proBNP levels between 125-900 ng/L. PAF denotes the group of participants with pAF at screening. Control denotes the group of participants with NT-proBNP levels less than 125 ng/mL and no evidence of pAF at screening.
